# Development and validation of a dietary screener for carbohydrate intake in endurance athletes

**DOI:** 10.1186/s12970-018-0250-y

**Published:** 2018-09-14

**Authors:** Stéphanie Harrison, Élise Carbonneau, Denis Talbot, Simone Lemieux, Benoît Lamarche

**Affiliations:** 1 Institute of Nutrition and Functional Foods (INAF), Pavillon des Services, Laval University, 2440, Hochelaga Boulevard, Quebec City, G1V 0A6 Canada; 2 School of Nutrition, Laval University, Quebec, G1V 0A6 Canada; 3 Department of Social and Preventive Medicine , Laval University, Quebec, G1V 0A6 Canada

**Keywords:** Carbohydrates, Dietary screener, Endurance athletes

## Abstract

**Background:**

Studies have shown that the majority of endurance athletes do not achieve the minimal recommended carbohydrate (CHO) intake of 6 g/kg of body weight (BW), with potentially negative impacts on recovery and performance. The purpose of this study was to develop and validate a rapid and easy to use dietary screener to identify athletes who do and do not achieve a CHO intake > 6 g/kg BW in the context of endurance sports.

**Methods:**

The dietary screener was developed using multiple logistic regression modeling of data from a sample of 1571 non-athlete adults (826 women and 745 men, mean age 44.75 ± 14.2 years) among whom dietary intake was assessed using a validated web-based food frequency questionnaire (web-FFQ). Three models were developed based on whole food intake using the 5, 10 and 15 most significant variables predicting CHO intake. The three models were then validated in a target population of non-elite endurance athletes having taken part in multisport events (*n* = 175, 64 women and 111 men, mean age 37.1 ± 11.3 years) and compared using sensitivity, specificity, positive and negative predictive values (PPV and NPV, respectively) and c-statistics.

**Results:**

The 15-variables model provided significantly better accuracy in predicting CHO intake adequacy in non-elite endurance athletes (c-statistic = 0.94) compared with the 10- and 5-variables model (c-statistic = 0.90 and 0.71 respectively). The 15-variables model predicts CHO intake adequacy in the target population of endurance athlete with a sensitivity of 89.5%, a specificity of 87.3% and PPV and NPV of 77.3 and 94.5%, respectively.

**Conclusion:**

We have successfully developed a short and valid dietary screener that identifies endurance athletes at risk of not achieving a CHO intake > 6 g/kg BW. Use of this rapid screener may help alleviate the highly prevalent issue of suboptimal CHO consumption in the endurance sports realm.

## Background

Carbohydrates (CHOs) are a crucial component of an athlete’s diet, especially in endurance sports. Dietary CHOs contribute to restoring muscle and liver glycogen between training sessions and increase performance when their availability is maintained during the effort [1, 2]. Recommendations on dietary CHOs are specific to sport, training regimen and competition schedule [2]. It is generally recommended that athletes consume between 6 and 10 g of CHO per kg of body weight (BW) per day when involved in an endurance program comprising moderate-to-high intensity trainings [1].

Multiple studies have shown that a large proportion of endurance athletes do not meet these recommendations. For instance, 45% of non-elite men and women participating in endurance multisport events such as IRONMAN triathlons were below the targeted 6 g CHO/kg of BW [3]. The prevalence of inadequate CHO intake was 80% among elite endurance athletes [4]. Studies have also shown that average CHO consumption among young pentathlon athletes and female collegiate athletes was below the recommendation [5, 6]. This is an important concern because inadequate CHO consumption may lead to decreased work rates, impaired skills and concentration and increased effort perception, all of which are partly caused by fatigue [2]. Weakening of the immune system and increased risks of complications due to over-training have also been associated with low CHO intake in endurance athletes [7].

The difficulty in rapidly measuring food and nutrient intakes on the field certainly represents one of the most significant barriers to more optimal management of diet among athletes. Dietary assessment is costly and time-consuming and this is particularly troublesome in environments that generally rely on limited resources towards nutrition support. Having access to a rapid and cost-effective screening tool that identifies athletes at risk of not achieving dietary CHO recommendations will prove to be extremely useful to assign the limited nutrition support resources to those who need it the most. Therefore, the purposes of this study were 1) to develop a rapid and simple dietary screener predicting a CHO intake > 6 g/kg of BW, which is considered the minimal recommendation for several endurance sports during moderate-intense training periods and 2) to validate and test the predictive value of the screener in a sample of endurance athletes. The screener was developed in a large sample of non-athletes to maximize statistical power and hence the stability of the predictive model. While we recognize that the CHO needs of a non-athlete population are very different than those of endurance athletes, we worked on the premise that the predictors of a high CHO intake are essentially the same in the two populations. We hypothesized that it is possible to identify endurance athletes at risk of not consuming adequate amounts of CHO based on a simple screening tool.

## Methods

### Study participants

A database of adult non-athlete subjects from previous projects conducted at the Institute of Nutrition and Functional Foods (INAF) in Quebec City was used to develop the screener (DEV sample). Multiple projects, in which subjects were all healthy, were included in the database. All participants provided consent in written form to have their data included in a database for use in research other than the main project to which they participated. The validity of the screener in the targeted population was assessed in a sample of non-elite endurance athletes (VALID sample). These athletes competed in Ironman triathlons (IM), Ironman 70.3 triathlons (IM 70.3), winter pentathlon (tandem or solo category) (9–15 km of cycling, 3.6–5.5 km of running, 4.9–8 km of cross-country skiing, 5–8.4 km of ice-skating, and 3.4–5.1 km of snowshoeing) or winter triathlon (5 km of snowshoeing, 12 km of ice-skating, and 8 km of cross-country skiing). Non-elite athletes provided consent through an online system.

### Dietary data collection

Participants in both the DEV and the VALID samples completed a validated web-based food frequency questionnaire (web-FFQ) [8]. This questionnaire contains 136 questions split into eight different sections: dairy products, fruits, vegetables, meat and alternatives, cereals and grain products, beverages, ‘other foods’ and dietary supplements. The web-FFQ inquires about food intake during the month prior to questionnaire completion. The Nutrition Data System for Research (software version 4.03, Food and Nutrient Database 31, Minneapolis, MN, USA) (Schakel et al., 1988) and the Canadian Nutrient File (CNF, version 2007b, Ottawa, ON, Canada) (Health Canada, 2007) were used to obtain nutrient intakes based on the answers provided in the web-FFQ. Food items from a broad category were grouped as one variable to simplify application and use of the final screener. For example, answers pertaining to brown and white rice consumption were added together to form only one category (frequency and amount of rice consumption).

### Model development

Logistic regression modeling was used to develop the CHO-specific screener using data from the DEV sample. Analyses were undertaken in SAS (University Edition) unless stated otherwise. *P-values* less than 0.05 were considered statistically significant. Food servings per day, derived from the frequency and portion size of food categories in the web-FFQ, and participants’ sex were considered in the primary phases of model development. Intake of individual macro- and micronutrients, such as lipids or proteins, was not considered because not all endurance athletes are aware of their specific nutrient consumption. Indeed, those at the higher end of the performance spectrum may be more meticulous in monitoring their dietary intake, hence having the knowledge and the resources to assess their own CHO intake. A screener for CHO intake is therefore not targeting these athletes, but rather those with limited resources and knowledge, who are less likely to monitor their diet and have a sense of their own CHO intake. Finally, dietary supplements were not considered, as data on sport-specific supplements were not available in the DEV sample.

Spearman’s correlations between food intake (in servings/d) and CHO consumption (in g/kg of BW) were first calculated. The 25 foods showing the strongest univariate correlation with CHO consumption were retained. Then, for each of these 25 foods, cut-off points that best correlated with CHO intake above or below 6 g/kg BW were identified using logistic regression in R (version 3.3.0), in order to create dichotomic variables that can be answered simply by yes/no. These cut-off points were further adjusted to best reflect plausible daily or weekly servings. For example, the cut-off point for bread that best predicted a CHO intake > 6 g/kg of BW in univariate logistic regression was 2.67 servings/d. This value was rounded up to 3 servings per day in order to facilitate the answering of the question by athletes. Next, multiple stepwise logistic regression models were constructed in SAS based on the 5, 10 and 15 variables that best predicted a CHO consumption > 6 g /kg of BW. Contingency tables with derived sensitivity, specificity, positive and negative predictive values (PPV and NPV, respectively) as well as receiver operating characteristic (ROC) curves and derived c-statistic were used to compare the performance of the three models. A model based on 20 variables or more was considered, but data indicated that model performance was no longer increased beyond 15 variables (not shown).

### Model validation

Model validation was ascertained in the VALID sample of non-elite endurance athletes. The 5, 10 and 15-variables models derived from the development phase were compared for performance again using statistics from contingency tables as well as ROC curves.

## Results

### Participants characteristics

The DEV sample included 1571 participants (826 women and 745 men). Mean age was 44.8 years (SD = 14.3), mean body mass index (BMI) was 28.1 kg/m^2^ (SD = 5.8) and mean CHO consumption was 3.75 g/kg of BW (SD = 1.5). Only 7.2% of participants in this sample had a CHO consumption > 6 g/kg of BW (Table 1).

**Table 1:** Characteristics of subjects in the DEV sample (*n* = 1571)

Women, %	52.6%
Age, *y*	44.8 ± 14.3^a^
Body weight, kg	79.9 ± 18.9
BMI, kg/m^2^	28.1 ± 5.8
Carbohydrates consumption, g/kg of body weight	3.75 ± 1.5
Subjects consuming > 6 g CHO/kg of body weight, (%)	7.2%

^a^Mean ± SD (all such values) unless stated otherwise

The VALID cohort included 175 athletes (64 women and 111 men). Mean age was 37.1 years (SD = 11.3), mean BMI was 23.3 kg/m^2^ (SD = 2.6) and mean CHO consumption was 5.4 g/kg BW (SD = 2.5). A total of 32.6% consumed more than 6 g of CHO/kg of BW (Table 2).

**Table 2:** Characteristics of subjects in the VALID sample (*n* = 175)

Women, %	36.6%
Age, *y*	37.1 ± 11.3^a^
Body weight, kg	69.1 ± 11.1
BMI,^b^ kg/m^2^	23.3 ± 2.6
Carbohydrates consumption, g/kg of body weight	5.4 ± 2.5
Subjects consuming > 6 g CHO/kg of body weight, %	32.6%

^a^Mean ± SD (all such values) unless stated otherwise

^b^*n =* 147 because of 28 missing height values

### Model development

**Fig. 1:**
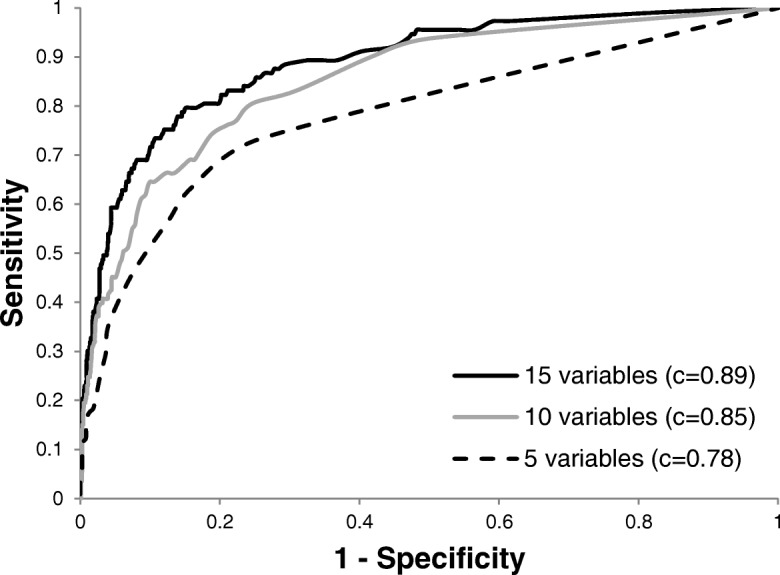
ROC curves comparison of multiple logistic regression models in DEV sample. (*c* represents the c statistic on a scale of 0.5 to 1.0)

The 15-variables model showed the highest c-statistic (0.89, *p* < 0.004 vs other models, Fig. 1) with a sensitivity of 73.5%, a specificity of 86.7%, a NPV of 97.7% and a PPV of 30.0% (Table 3).

**Table 3:** Characteristics of the multiple logistic regression models in the DEV sample

Model	Sensitivity	Specificity	False positives	False negatives	PPV	NPV	*c* statistic
5 variables^a^	63.7	83.8	70.0	2.3	23.4	96.8	0.78
10 variables	64.6	87.7	71.0	3.0	29.0	97.0	0.85
15 variables	73.5	86.7	70.0	2.3	30.0	97.7	0.89

*PPV* positive predictive value, *NPV* negative predictive value

^a^ % (all such values)

### Model validation

**Fig. 2:**
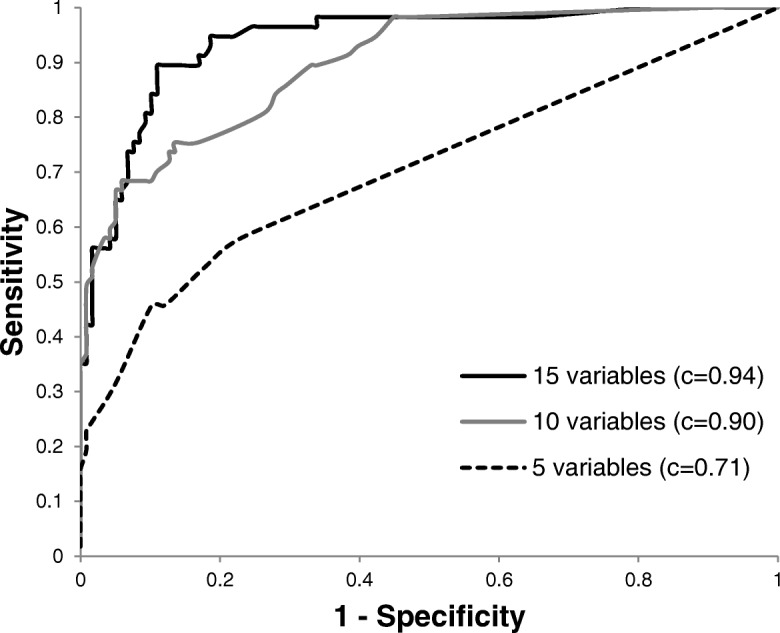
ROC curves comparison of multiple logistic regression models in the VALID sample. (*c* represents the *c* statistic on a scale of 0.5 to 1.0)

Table 4 shows the characteristics of the 5, 10 and 15-variables models when applied to the target population of endurance athletes (VALID cohort), using the predetermined cut-offs for each food in the model. Consistent with data from the DEV sample, the 15-variables model performed significantly better than the 5 and 10-variables models in predicting CHO intake (Fig. 2). The 15-variables model identified athletes achieving the minimal CHO recommendation (> 6 g/kg of BW) with a sensitivity of 89.5% and a specificity of 87.3%. NPV and PPV were 94.5 and 77.3%, respectively. Table 5 presents the final screener based on the 15-variables model, presenting each food retained in the model with its corresponding cut-off (formulated as a question) and their associated multivariate ß derived from the multivariate logistic model. These ß are used to define the predictive model that will be deployed to predict one’s risk of not achieving the CHO recommendations for endurance sports.

**Table 4:** Characteristics of the multiple logisitic regression models in VALID sampler

model	Sensitivity	Specificity	False positives	False negatives	PPV	NPV	*c* statistic
5 variables^a^	52.6	82.2	12.0	15.4	58.8	78.2	0.71
10 variables	75.4	86.4	9.1	8.0	72.9	87.9	0.90
15 variables	89.5	87.3	8.6	3.4	77.3	94.5	0.94

*PPV* positive predictive value, *NPV* negative predictive value

^a^ % (all such values)

**Table 5:** Final dietary screener

Questions of the final dietary screener^a^	β흱^b^
Do you consume melons (watermelon, honeydew or cantaloup) on a daily basis?	0.5287
Do you consume pancakes twice a week?	1.9666
Do you consume avocado twice a week?	−0.0433
Do you consume cereal bars 6 times a week?	2.0899
Do you consume rice 5 times a week?	2.0401
Do you drink chocolate milk 5 times a week?	2.3249
Do you consume chocolate (white, milk or dark) every week?	1.8776
Do you consume corn on a daily basis?	0.7994
Do you consume milk, soy milk or silk tofu based desserts 3 times a week?	5.3373
Do you consume cold breakfast cereals on a daily basis?	3.0771
Do you consume pasta on a daily basis?	0.8276
Do you consume jam, maple by-products, hazelnut spread, jelly or chocolate syrup twice a day?	2.3477
Do you consume salad, lettuce or spinach twice a day?	2.7638
Do you drink soft drinks 3 times a day?	10.3662
Are you a woman?	0.3734

^a^ Final questions are based on optimal cut-off points calculated by R (version 3.3.0) that were further adjusted to best fit a daily or weekly number of servings. Cut-off points represent the number of servings of each specific food that best predicted a CHO consumption > 6 g/kg of BW

^b^β from the multivariate logistic regression model for each dichotomic variable (yes/no) in the final dietary screener. All β are significant (*P* < 0.05)

## Discussion

Rapid and cost-efficient assessment of CHO consumption among endurance athletes is challenging on the field. Although multiple dietary assessment tools, such as FFQs, 24 h recalls and dietary journals, are available to calculate an athlete’s CHO intake, these tools usually take a lot of time to complete and require the experience of a trained professional for analysis. This, combined with the fact that large proportions of endurance athletes do not meet the recommended CHO intake, is a concerning issue. Here, we have developed a CHO-specific dietary screener that allows rapid detection of endurance athletes at risk of not achieving a CHO intake of 6 g/kg of BW or more. To our knowledge, this is the first validated tool that screens for adequate CHO intake among athletes.

The final model upon which the screener is based has both a high sensitivity and specificity in the target population (89.5 and 87.3%, respectively), which are desired traits [9]. Such statistics indicate that the screener is as accurate in adequately identifying athletes who meet and those who do not meet the recommendation for CHO intake. The high AUC of the ROC curve (or c-statistic) yielded by the 15-variables model (0.94 on a range from 0.5 to 1.0) is also reflective of a dietary screener that has excellent accuracy [9]. Furthermore, the model’s NPV was considerably higher than its PPV (94.5% vs. 77.3%), indicating that the screener is slightly more accurate in identifying endurance athletes who do not achieve adequate CHO intake than those who do. Such characteristic is highly desirable in the context of this research, as the ultimate goal of the CHO screener is to target athletes who would benefit from nutritional counseling, i.e. those with inadequate CHO intakes.

Very few studies have used an approach similar to ours to develop predictive models of adequate/inadequate dietary intakes, which makes comparison difficult. Most attempts were undertaken with a health rather than sports perspective. In those previous studies, predictive models and tools often achieved either a high sensitivity or a high specificity, but rarely both. For instance, Cook et al. built single-question and five-question screeners to rapidly assess fruits and vegetables intake among non-athletes. Sensitivity values ranged from 35.7 to 45.5% while specificity values ranged from of 81.8 to 84.9% among all five-question screeners developed by the research team. Using a single-question approach yielded high sensitivity but low specificity in the same population [10]. In most of these studies, the AUC of the ROC curves were fairly low, indicative of poor accuracy.

In an attempt to develop the simplest and yet most accurate CHO screener possible, we gave important considerations to limitations specific to the sports work environment. First, we had access to numerous dietary variables for the development of the model, such as energy, vitamin and protein intake, which may have contributed to a better prediction accuracy. However, such information is not readily available to either the respondent or the resource responsible for the screening test. It was therefore decided a priori to exclude such information. All anthropometric measures were also a priori excluded as they are too-closely related to the outcome measure to predict, which is based on BW. Similarly, cut-off values for each predictive food in the model were rounded to full daily or weekly servings to facilitate screener administration.

Several methods can be used to develop the predictive model of an outcome. Here, a multifaceted approach was used, but ultimately a stepwise logistic regression modeling approach yielded the final model. A classification tree (CT) approach was also considered to develop the screener. This method uses discriminant analysis to test various combinations of variables in order to maximize the CT’s predictive power [11]. Different algorithms can be used to build CTs; the CART algorithm was the chosen method for our purpose. What characterizes the CART algorithm is that it builds a very large CT and then prunes it to a smaller size to minimize classification errors [11]. A 10-fold cross-validation is used to prune the initial CT. The use of this method would have been beneficial for this particular research since ideal cut-off points are calculated directly in the CT algorithm. Unfortunately, this method yielded underwhelming results, with unacceptably high values for false negatives (approximately 30%) when applied to the athletes sample (the VALID cohort). We hypothesize that the sample of non-athletes used to develop the CT may have comprised too few individuals with a CHO intake > 6 g/kg of BW, thereby reducing the data usable by the algorithm to maximize the CT’s predictive power.

Although this is the first study to develop a CHO-specific dietary screener for endurance athletes, limitations should be noted. First and foremost, the sample used to build the screener for application among athletes comprised non-athletes. This may have been a very significant shortcoming, considering that the diets of non-athletes and of endurance athletes are quite different. Second, a small proportion of the sample of individuals used to develop the screener achieved an intake of CHO greater than 6 g/kg of BW, which may have hindered our ability to accurately predict this nutritional outcome. Ideally, the development of this CHO-specific screener would have been based on data from a large cohort of endurance athletes, but this was not possible. Third, the target of 6 g CHO/kg BW may not be applicable to every endurance sport or training regimen, and this is a limitation when using the screener among athletes whose CHO needs are greater than 6 g/kg of BW. Furthermore, participants in the development sample were not asked about CHO supplements often used in endurance sports. This is a limitation of the screener as intake of CHO is influenced by the use of such supplements in athletes. Nevertheless, the accuracy and hence validity of the CHO-specific screener among endurance athletes is considered to be excellent, despite these limitations. Lastly, exploring different approaches for model development is a strength considering that very few studies in the field of nutrition have used CTs to create predictive models.

## Conclusion

In sum, we were successful in developing a simple, 15-questions dietary screening tool that predicts with accuracy an athlete’s risk of achieving a dietary CHO target of 6 g/kg BW for endurance sports. Since the screener was validated in non-elite endurance athletes, further research should be conducted to test the accuracy of the screening tool among elite endurance athletes. The extent to which information on supplement use, particularly CHO supplements, improves prediction of total CHO intake by the screener needs to be investigated in future studies. Nevertheless, this easy-to-use screening tool will be a great asset to field work in sports nutrition as it rapidly identifies athletes who may benefit the most from receiving dietary counseling to optimize their diet.

## Abbreviations

AUC: Area under the curve
BW: Body weight
CHO: Carbohydrate
CNF: Canadian nutrient file
CT: Classification tree
IM: IRONMAN triathlon
IM70.3: IRONMAN 70.3 triathlon
INAF: Institute of Nutrition and Functional Foods
NPV: Negative predictive value
PPV: Positive predictive value
ROC: Receiver operating characteristic
Web-FFQ: Web-based food-frequency questionnaire
